# ACCEPTABILITY OF A VIDEOCONFERENCE-BASED MULTICOMPONENT EXERCISE PROGRAM (VIVIFRAIL) FOR THE OLDEST-OLD

**DOI:** 10.1093/geroni/igac059.980

**Published:** 2022-12-20

**Authors:** Ana Tiecker, Angelo Jose Bos

**Affiliations:** PUCRS, Porto Alegre, Rio Grande do Sul, Brazil; Pontifical Catholic University of Rio Grande do Sul, PORTO ALEGRE, Rio Grande do Sul, Brazil

## Abstract

**Introduction:**

Previous studies have shown the effectiveness of supervised multicomponent exercise programs on counteract age-related changes in functional capacity and quality of life in the oldest-old. During the quarantine imposed by COVID-19 pandemic, social isolation has been a barrier to implement face-to face exercise programs. Objective: To study the acceptance and adequacy of an exercise protocol in oldest-old. Methodology: Quasi-experimental study with initial, intermediate and post-intervention evaluation. The functional capacity assessment was carried out by the Vivifrail test (Short Physical Performance Battery and 4 falls risk assessment tests), by videoconference at the three moments of the project. For twelve weeks, the older adults performed multicomponent ViviFrail exercises with monitoring and acceptance assessment (with a maximum score of 24 points) by weekly contact by videoconference.

**Results:**

This study concluded 14 oldest-old (89.07 ± 6.30 years). In the 12 weeks, the participants showed an average increase of 4.2 points in acceptance, with a significant correlation (p < 0.001). It was possible to observe an improvement in the functional capacity of the oldest old, although not significant, with a decrease in the time to perform the Time Up and Go tests, sit and stand, and in walking time, which is the most evident change.

**Conclusions:**

This study demonstrated that a home training program with weekly monitoring by videoconference was well accepted and suitable for oldest-old people in a period of social isolation imposed by COVID-19. In addition, it proved to be an effective intervention for maintaining and improving the functional capacity of oldest-old people.

